# Genomic Signatures Associated with Transitions to Viviparity in Cyprinodontiformes

**DOI:** 10.1093/molbev/msad208

**Published:** 2023-10-04

**Authors:** Leeban H Yusuf, Yolitzi Saldívar Lemus, Peter Thorpe, Constantino Macías Garcia, Michael G Ritchie

**Affiliations:** Centre for Biological Diversity, School of Biology, University of St Andrews, St Andrews, UK; Centre for Biological Diversity, School of Biology, University of St Andrews, St Andrews, UK; Department of Biology, Texas State University, San Marcos, TX, USA; The Data Analysis Group, School of Life Sciences, University of Dundee, Dundee, UK; School of Medicine, University of North Haugh, St Andrews KY16 9TF, UK; Instituto de Ecologia, Universidad Nacional Autónoma de México, Ciudad Universitaria, Mexico City CdMx, Mexico; Centre for Biological Diversity, School of Biology, University of St Andrews, St Andrews, UK

**Keywords:** convergent evolution, molecular evolution, comparative genomics, viviparity

## Abstract

The transition from oviparity to viviparity has occurred independently over 150 times across vertebrates, presenting one of the most compelling cases of phenotypic convergence. However, whether the repeated, independent evolution of viviparity is driven by redeployment of similar genetic mechanisms and whether these leave a common signature in genomic divergence remains largely unknown. Although recent investigations into the evolution of viviparity have demonstrated striking similarity among the genes and molecular pathways involved across disparate vertebrate groups, quantitative tests for genome-wide convergent have provided ambivalent answers. Here, we investigate the potential role of molecular convergence during independent transitions to viviparity across an order of ray-finned freshwater fish (Cyprinodontiformes). We assembled de novo genomes and utilized publicly available genomes of viviparous and oviparous species to test for molecular convergence across both coding and noncoding regions. We found no evidence for an excess of molecular convergence in amino acid substitutions and in rates of sequence divergence, implying independent genetic changes are associated with these transitions. However, both statistical power and biological confounds could constrain our ability to detect significant correlated evolution. We therefore identified candidate genes with potential signatures of molecular convergence in viviparous Cyprinodontiformes lineages. Motif enrichment and gene ontology analyses suggest transcriptional changes associated with early morphogenesis, brain development, and immunity occurred alongside the evolution of viviparity. Overall, however, our findings indicate that independent transitions to viviparity in these fish are not strongly associated with an excess of molecular convergence, but a few genes show convincing evidence of convergent evolution.

## Introduction

The extent of genomic homology during the convergent evolution of complex phenotypes is a major question in evolutionary genetics but poorly resolved. Although the independent acquisition of similar traits in lineages that do not share recent common ancestry is eloquent evidence for parallel adaptive evolution, such phenotypic convergence may utilize similar genetic changes (Castoe et al. 2009; Martin and Orgogozo 2013; Stern 2013; Natarajan et al. 2015), but sometimes distinct molecular and developmental processes are involved (Steiner et al. 2008; Berens et al. 2014; Natarajan et al. 2016; Murugesan et al. 2022). Recent attempts to understand the genetic bases of phenotypic convergence have demonstrated the importance of comparative phylogenomic approaches to identify genetic changes responsible for independent trait transitions across different taxonomic groups (Smith et al. 2020). Even when common genetic factors are involved, they may be diverse, ranging from parallel amino acid changes (Johanson et al. 2000; Sugawara et al. 2005; Kuittinen et al. 2008), gene family evolution (Christin et al. 2007), convergent sequence divergence in protein-coding genes (Chikina et al. 2016), and convergent sequence divergence in regulatory regions (Sackton et al. 2019). However, disentangling confounding factors from convergent evolution have proven difficult (Parker et al. 2013; Thomas and Hahn 2015; Zou and Zhang 2015). The challenge is often exacerbated in cases of complex phenotypic traits, where convincing signals of molecular convergence may be especially difficult to detect (Corbett-Detig et al. 2020; Yusuf et al. 2020), and previous comparative genomic analyses that have searched for convergence have been limited as they either 1) focus only on convergent amino acid changes, 2) are restricted to convergent shifts in gene-wide evolutionary rate, or 3) lack empirical null models to determine whether neutral processes may explain the observed signals of molecular convergence. Few attempts to characterize molecular convergence have jointly addressed the potential role of phylogenetic incongruence and hemiplasy in confounding patterns of molecular convergence (Sackton et al. 2019; Corbett-Detig et al. 2020; Hibbins, Gibson and Hahn 2020).

The convergent evolution of viviparity provides a tractable system to explore complex, independent trait transitions, and the consistency of molecular adaptations. Viviparity has independently evolved from oviparity (egg laying) more than 150 times across diverse vertebrate groups, with fish and squamate reptiles accounting for most transitions, which include several potential reversals to oviparity that have received mixed phylogenetic support (Surget-Groba et al. 2006; Cornetti et al. 2014; Blackburn 2015; Recknagel et al. 2018; Horreo, Suarez and Fitze 2020; Whittington et al. 2022). In some reproductively bimodal squamates, intraspecies variation in parity is observed in the form of intermediate, genetically determined phenotypes (Smith and Shine 1997; Qualls and Shine 1998; Horreo et al. 2020; Recknagel et al. 2021). Elucidating the genetic changes involved at a population and phylogenetic scale is complicated by the fact that the evolution of viviparity involves a suite of complex morphological, physiological, and developmental changes including egg retention, internal fertilization, immunotolerance, internal embryonic development, and nutrient and gas exchange (Van Dyke et al. 2014). These multiple adaptations are in part explained by the maternal–fetal interface and degree of maternal investment, which may differ considerably among viviparous species, from development depending exclusively on the nutrients deposited on the egg yolk (lecithotrophy), to embryos relying on a constant supply of maternal nutrients during development (matrotrophy), or somewhere in between extremes of this continuum (Blackburn 1992).

Previous analyses of the genetics of viviparity have largely focused on genomic and transcriptomic transitions linked to mammalian viviparity, despite it only containing a single transition in reproductive mode (Lynch and Wagner 2008; Lynch et al. 2008; Emera et al. 2012; Kin et al. 2016; Nnamani et al. 2016). Recent analyses in lizards (Whittington et al. 2015) as well as seahorses and pipefish (the last two of which have repeatedly evolved what some authors have called paternal pregnancy; Gao et al. 2019; Roth et al. 2020) have identified modules of genes that show homology with pregnant female mammals, suggesting they may be crucial genetic components in vertebrate transitions to viviparity and internal gestation within the body of any of the parents. In particular, shifts in the expression of genes involved in hormonal regulation, tissue remodeling, and nutrient exchange, as well as convergent loss of genes involved in adaptive immune response, were found to coincide with the evolution of internal gestation inside the father's body in these taxa. However, there have been few explicit tests of genome-wide molecular convergence linked to viviparity and internal gestation and associated adaptations, such as the placenta or placentalike structures, outside of mammals (but see: Gao et al. 2019; Roth et al. 2020; Recknagel et al. 2021; van Kruistum et al. 2021). In squamates, assessing gene expression differences in the oviducts of oviparous and viviparous species (Gao et al. 2019), as well as pregnant and nonpregnant states (Recknagel et al. 2021), has demonstrated large-scale expression changes across many genes involved in eggshell reduction, placentation, nutrient transport, and embryogenesis which are associated with the transition to viviparity but with no clear role for convergent protein-coding sequence evolution. In viviparous fish, convergent genomic changes in coding sequences are associated with turnover of immune-related gene repertoires (Roth et al. 2020), coding and noncoding changes are associated with the evolution of placentation (van Kruistum et al. 2021), and gene expression changes known to be important in mammalian viviparity and “male pregnancy” in seahorses and pipefish are also associated with the evolution of viviparity in Goodeinae (Du et al. 2022). In contrast, Foster et al. (2022) analyzed the transcriptomes of the placenta of eight vertebrates and concluded that there was no significant overlap in gene involvement, so each placenta had evolved from unique changes in gene expression networks.

Cyprinodontiformes is an order of small, freshwater ray-finned fish that show considerable diversity in reproductive mode and associated adaptations (Wourms and Lombardi 1992). Within the order, the evolution of viviparity has been linked to bursts of diversification explained by changes in associated life history traits, sexual selection, and sexual conflict (Helmstetter et al. 2016). For example, in poecilids, matrotrophy and placentotrophy, the reliance on maternal provisioning via placenta postfertilization, has evolved independently multiple times from a nonplacental ancestor (Pollux et al. 2009; Furness et al. 2019). Alongside lecithotrophy, where nutrients are supplied to offspring prefertilization, poecilids also demonstrate variation in placental complexity and the degree of provisioning in matrotrophy (Pollux et al. 2009). Phylogenetic comparative methods have demonstrated that this transition from lecithotrophy to matrotrophy and placentation is associated with sexual conflict and sexual selection in poecilids (Pollux et al. 2014; Furness et al. 2019). Similarly, comparative genomic analyses within the order have specifically focused on understanding the genetic context of placental evolution, uncovering parallels with mammalian viviparity and the independent acquisitions of placentae in poecilids (Guernsey et al. 2020; van Kruistum et al. 2021).

The Goodeidae, a family of mostly viviparous ray-finned fish within the order Cyprinodontiformes, have received less attention. The Goodeinae are proposed to have become viviparous after diverging from oviparous species of the family in the early Miocene, before subsequent diversification of matrotrophic adaptations (Webb et al. 2004). Across goodeins, matrotrophy is prevalent and is linked to the evolution of a novel, placental analog consisting of a maternal component, the internal ovarian epithelium, and an embryonic component, the trophotaenia (Lombardi and Wourms 1985; Wourms et al. 1988; Schindler 2015). Nutrients provided by the mother are absorbed via the trophotaenia and fuel rapid embryonic growth following early embryonic development (Schindler 2003). Additionally, there is interspecific variation in the degree of matrotrophy and sexual dimorphism across the group (Garcia et al. 1994; Moyaho et al. 2004; Garcia and Ramirez 2005; Ritchie et al. 2007), indicating the potential importance for sexual selection and conflict. However, the role of sexual selection and conflict as a driver of viviparity and matrotrophy in Goodeinae remains unclear (Ritchie et al. 2005; Saldivar Lemus et al. 2017).

Here, we utilize de novo genome assemblies and publicly available genomes to identify genomic features associated with the evolution of viviparity. Specifically, we use a phylogenomic comparative framework consisting of poecilids from four different genera (*Poecilia*, *Gambusia*, *Poeciliopsis*, and *Xiphophorus*) and goodeids from two subfamilies (Goodeinae and the Empetrichthyinae), alongside oviparous pupfish (*Cyprinodon* and *Orestias*) and mummichog (*Fundulus*) genomes. Our phylogenetic framework is thought to contain two independent transitions from oviparity to viviparity (Helmstetter et al. 2016). By comparing 16 viviparous and 5 oviparous genomes and leveraging their phylogenetic context, we sought to identify genomic regions associated with the convergent evolution of viviparity in Cyprinodontiformes at three levels by assessing: 1) convergent changes in amino acids, 2) evolutionary rate change across protein-coding genes, and 3) evolutionary rate change in conserved, noncoding regions. Additionally, to test whether genome-wide convergent amino acid changes and evolutionary rate divergence could be explained by neutral processes, we computed empirical null models by randomizing and retesting foreground branches in our phylogenetic framework. As with some previous analyses looking for quantitative evidence of convergent molecular changes (Thomas and Hahn 2015; Zou and Zhang 2015; Gao et al. 2019; Corbett-Detig et al. 2020), we find no evidence of an excess of genome-wide convergence in protein-coding genes that may explain transitions to viviparity. We do identify genes associated with shifts to viviparity and discuss how detecting signals of convergence may be confused by factors such as incomplete lineage sorting (ILS).

## Methods

### Sample Collection

Muscle tissue samples were obtained from adult males of *Goodea atripinnis*, *Xenotaenia resolanae*, *Xenophoorus captivus*, and *Crenichthys baileyi*. *Goodea atripinnis*, *X. resolanae*, and *X. captivus* individuals were descendants of fish captured in Michoacán, Jalisco, and San Luis Potosí states (Mexico) under SEMARNAT permit SGPA/DGVS/00824/20. The genome of a male of the oviparous goodeid *C. bailey*i was obtained from Kees de Jong.

### Whole-Genome Sequencing

Extracted DNA was sent to Novogene (Beijing, China), for library preparation and sequencing. Sequencing libraries were generated using the NEBNext DNA Library Prep Kit (New England Biolabs, USA) following the manufacturer's instructions. The genomic DNA was sheared to a size of 350 bp, and then, the fragments were end-polished, A-tailed, and ligated with the NEBNext Adapter (New England Biolabs, USA) for Illumina sequencing. Resulting libraries were analyzed for size distribution with 2100 Bioanalyzer (Agilent) and quantified using real-time polymerase chain reaction (PCR). Paired-end sequencing was performed on an Illumina NovaSeq 6000 system (Illumina Inc.) using the v1.0 reagents for sequencing.

### Genome Assembly

Raw reads were interrogated for quality using FastQC (Andrews 2010) and then quality trimmed using Trimmomatic (version 0.38) (Q15) (Bolger et al. 2014). The quality-controlled reads were then assembled using SPAdes (version 3.14.1) (Prjibelski et al. 2020). BlobTools version 1.0 (Laetsch and Blaxter 2017) was used to identify and remove contaminant contigs; the assembly was compared with the nonredundant database (GenBank nt) using BlastN (MegaBLAST). Contigs identified as fungal, bacterial, plantal, or viral were removed, yielding a contamination-free final unpolished assembly. The assemblies were then scaffolded using one iteration SSPACE (v3.0) (Boetzer et al. 2011) with Burrows–Wheeler Alignment (BWA). Finally, 3 iterations of Pilon (version 1.23) (Walker et al. 2014) was performed to polish the assembly. At all stages of assembly Benchmarking Universal Single-Copy Orthologs (BUSCO) (Simão et al. 2015) version 1.1b was used to assess the relative completeness of the assembled genomes using Eukaryotic odb9 models.

### Genome Alignment and Filtering

**Fig. 1.**
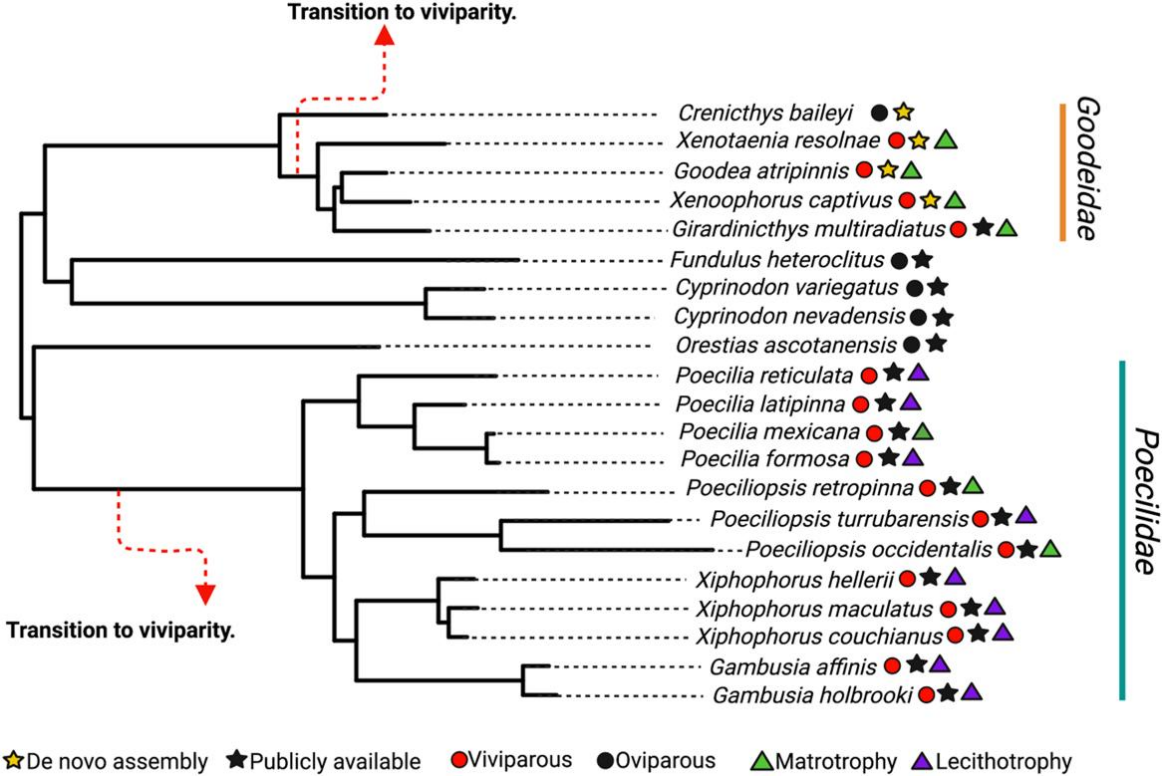
Pruned phylogenetic tree of the order Cyprinodontiformes. This is the phylogeny used in comparative analysis of molecular convergence. Both hypothesized transitions to viviparity used as foreground branches in the analysis are shown, alongside character states (either viviparous or oviparous) for each species denoted by circles near species names. Stars denote whether genomes were assembled here or whether they were publicly available. Triangles denote provisioning strategy.

Additional genomes of closely related viviparous and oviparous Cyprinodontiformes species (fig. 1) were downloaded from RefSeq and GenBank (supplementary table S1, Supplementary Material online). To determine evolutionary relationships and divergence times, a time-calibrated phylogeny for Cyprinodontiformes was retrieved from Rabosky et al. (2018) and pruned using the ape (v5.5) package in R to retain the relevant species used here (Paradis et al. 2003; Paradis and Schliep 2019). To produce a whole-genome alignment of the 21 genomes, we aligned all genomes to a reference genome (*Girardinichthys multiradiatus*; Du et al. 2022) using LAST aligner (Kiełbasa et al. 2011; Hamada et al. 2017), followed by chaining and netting using scripts and utilities from the UCSC browser source code (Miller et al. 2003). Finally, a multiple whole-genome alignment of the 21 species was built from the reciprocal-best nets using MULTIZ (Blanchette et al. 2004), with *G. multiradiatus* as the reference.

Protein-coding sequences were extracted from the multiple whole-genome alignment using the *G. multiradiatus* genome annotation (Du et al. 2022) and MafFilter (v1.3.0) (Dutheil et al. 2014). To obtain aligned orthologous amino acid sequences, we initially aligned coding sequences using MAFFT (v7.471) with default settings (Katoh et al. 2002, 2005; Katoh and Standley 2013; Nakamura et al. 2018). Subsequently, MACSE v.2 was used to mask frameshifts and stop codons and the resulting nucleotide sequences were converted into amino acid sequences (Ranwez et al. 2018). Finally, Divvier (options: partial -divvygap -mincol 21) was used to retain only clusters of high-confidence amino acid columns in alignments with evidence of shared homology, removing variable columns in alignments that were deemed low-confidence (Ali et al. 2019). For codon sequences, MACSE v.2 was used to trim homologous fragments from the beginning and end of alignments and then MACSE v.2 was used to align sequences. Codon alignments were translated into amino acids and subsequently converted into final codon alignments using PAL2NAL, specifying the removal of incomplete codons and gaps (Suyama et al. 2006).

### Phylogenetic Reconstruction of Species Tree

In order to infer species relationships, we retrieved 1,044 single-copy orthologs from a BUSCO analysis of all 21 species (performed using the Actinopterygii gene set) (Simão et al. 2015). Species-specific proteomes consisting of these single-copy orthologs were then used to infer orthogroups and a species tree using OrthoFinder2 and IQ-TREE2 with the following parameters: -M msa -T iqtree (Nguyen et al. 2015; Emms and Kelly 2018).

### Protein-Coding Genes Associated with the Transition to Viviparity

To detect convergent amino acid changes in lineages that have evolved viviparity, we utilized the TDG09 program, which compares a null model assuming homogenous substitution patterns for each site in the alignment across all branches in the phylogeny, with a model which assumes nonhomogenous substitution patterns between “foreground” branches where viviparity is assumed to have evolved and “background” branches (Tamuri et al. 2009). All amino acid alignments that passed filtering were tested using the species tree with “foreground” or “background” annotations ascribed to each species in the tree (groups VI OV, for viviparity and oviparity, respectively). To determine whether an excess of genome-wide convergent amino acid changes has occurred in foreground branches, empirical null models of the phylogenetic framework were conducted. Foreground branches were randomized in two different control tests, and all sites were retested. Only sites with a false discovery rate (FDR) < 0.05 (Benjamini and Hochberg 1995) were considered to be convergently evolving.

Convergent genetic changes may involve the same changes in single amino acids, but also coding sequences may show correlated divergence across the entire protein. We used RERconverge to test the correlation between relative rates of protein evolution and convergent trait evolution at foreground branches on the species tree (Chikina et al. 2016; Kowalczyk et al. 2019). We generated phylogenetic trees for all amino acid alignments using the LG amino acid matrix and by fixing the tree topology based on the species tree so that only branch lengths were estimated (Le and Gascuel 2008; Schliep 2011) and then transformed into relative evolutionary rates to determine whether, for a given branch, genes have evolved faster or slower than the background rate.

Branch-specific relative change rates were then used in a correlation analysis with binary trait classifications of either “viviparity” or “oviparity,” where viviparous species were designated as foreground branches. As a control, we randomly sampled two foreground branches across the phylogeny and retested all genes to determine whether we observed an excess of molecular convergence. A weighted correlation was used to correct for heteroscedasticity. Weighted correlations were carried out using Kendall's Tau and multiple testing correction was performed using the Benjamini–Hochberg procedure (Benjamini and Hochberg 1995).

To test whether genes showing an association with viviparity in our fish samples were expressed in mammalian placental tissue, expression data for mammalian placental tissues across 14 species were retrieved from Armstrong et al. (2017). To assess whether genes showing significant relative evolutionary rates (SRER) in foreground branches where viviparity evolved also showed higher than expected expression in the mammalian placenta, we compared mean expression (as fragments per kilobase per million reads [FPKM]) between SRER genes and an empirical null distribution generated by permuting (1,000 iterations) expression levels of randomly sampled genes. Additionally, expression data for *Poeciliopsis turrubarensis* (lecithotrophic) and *Poeciliopsis retropinna* (matrotrophic) were retrieved from Guernsey et al. (2020). Specifically, genes were listed as either expressed in the maternal follicles of *P. turrubarensis*, *P. retropinna*, or both.

### Testing for Evidence of Positive Selection and Shifts in Selection Pressure

To test for evidence of positive selection along viviparous lineages, we utilized two different codon substitution models: BUSTED and aBSREL (Murrell et al. 2015; Smith et al. 2015). Specifically, we tested for positive selection in genes that showed both at least one convergent amino acid substitution and a significantly different relative evolutionary rate in foreground branches. BUSTED tests for evidence of gene-wide positive selection—that is, whether there has been diversifying selection on at least one foreground branch and for at least one site in the alignment using the dN/dS ratio. An unconstrained model with three rate classes (which sites are assigned to) was compared with a null (constrained) model where diversifying selection is disallowed, using a likelihood ratio test. Alongside this, aBSREL was used to test if positive selection has occurred on a subset of sites in specified foreground branches, that is, a branch–site test. For both tests, foreground branches were specified as the branches where the hypothesized transition to viviparity occurred (fig. 1).

We also tested for positive selection on all internal and terminal branches leading to viviparous lineages that are not representative of the transition from oviparity to viviparity using aBSREL. We additionally used BUSTED to test for evidence of phylogeny-wide episodic selection (https://github.com/veg/hyphy-analyses/tree/master/BUSTED-PH) and specifically asked whether convergently evolving genes showed evidence of 1) episodic diversifying selection on foreground branches (our two branches of interest), 2) background branches, and 3) whether there was a difference in the distribution of foreground and background branches in terms of selective pressure. Finally, we used RELAX (Wertheim et al. 2015) to test whether, at branches where the transition to viviparity occurred (two foreground branches as observed in fig. 1), genes that show evidence of convergent evolution have experienced relaxation or intensification of selection.

Statistical analyses were conducted in R, and plots were produced using the “tidyverse” package (Wickham et al. 2019). Phylogenies were visualized using the ape package in R (Paradis et al. 2003; Paradis and Schliep 2019).

### Estimating the Probability of Hemiplasy and Accounting for Incomplete Lineage Sorting

Incomplete lineage sorting may potentially confuse signals of convergent evolution. To verify genuine signals of convergent evolution, we calculated gene concordance factors genome-wide, across gene sets showing evidence of convergent evolution, and performed reanalysis of molecular convergence analysis using plausible alternative topologies inferred from our gene concordance analysis. Gene concordance factors are defined as the percentage of gene trees that contain a given branch found in the species tree (Minh et al. 2020). To quantify phylogenetic discordance genome-wide, we calculated gene tree concordance factors using the species tree described above and gene trees inferred from 16,941 filtered amino acid alignment using IQ-TREE2 with the parameters: -m LG + G4 -nt 16.

To assess whether putatively convergently evolving loci may be disproportionately impacted by ILS, we calculated gene concordance factors on two subsets of our data: 1) genes with evidence of convergent amino acid changes and 2) genes with evidence of gene-wide convergent evolution. We compared phylogenetic gene discordance in these subsetted data sets to phylogenetic discordance across all genes predicting that ILS would lead to higher levels of discordance in convergently evolving genes identified using both approaches (1 and 2).

Additionally, we were interested in examining to what extent the fixed species tree topology used in our analyses may have affected our results. To do this, we utilized alternative tree topologies produced for each branch in our gene concordance analysis. We considered alternative topologies that differed predominantly at the two foreground branches of central interest, where the transition to viviparity likely occurred. In particular, two alternative topologies that differed at each of the two foreground branches of interest, respectively, were used to generate fixed topology gene trees where only branches were estimated using an LG model via phangorn (Le and Gascuel 2008; Schliep 2011). These two alternative topologies were the most well-supported alternative topologies for the transition to viviparity in 1) Goodeidae and 2) in Poecilidae, respectively. Subsequently, we reran our gene-wide convergent evolution analyses using RERconverge with these alternative topologies and assessed the overlap of genes showing evidence of convergent evolution when using the species tree topology compared with the other alternative, discordant topologies. Genes identified as evolving under convergent evolution under all three topologies are assumed to be robust to ILS.

We quantified the probability of hemiplasy in our phylogenetic framework using HeIST (Hibbins et al. 2020) which uses coalescent simulations to estimate the probability of hemiplasy and homoplasy under a multispecies network coalescent model, given a species tree and gene/site concordance factors. Specifically, we used the species tree inferred above with gene concordance factor estimates to convert branch lengths into smoothed coalescent units. Within HeIST, *ms* and *Seq-gen* were used to simulate 10^8^ loci along the species tree, and only focal loci reflecting the specific character states in the species tree were considered (Rambaut and Grassly 1997; Hudson 2002). Specifically, HeIST uses *ms* to simulate gene trees from a specified species tree and, subsequently, simulates the evolution of a nucleotide along each of these simulated gene trees using *Seq-gen*. Simulated loci with transformed nucleotide states (0/1 for ancestral or derived mutations, respectively) that match the character traits on the species tree (in this case, oviparous and viviparous) are considered focal loci. Since mutation rates for species within the phylogeny have not been estimated, we followed the rationale of Hibbins et al. (2020) and used a vertebrate-general estimate of 0.005 per 2*N* generations.

### Noncoding Elements Associated with Convergent Transition to Viviparity

In order to detect regulatory elements potentially underlying the transition from oviparity to viviparity, we extracted noncoding regions from our whole-genome alignment. We then partitioned the whole-genome alignment by scaffold using WGAbed (https://henryjuho.github.io/WGAbed/). To infer conserved noncoding genomic regions, we estimated a neutral model by extracting and using 4-fold degenerate sites across all scaffolds to transform branch lengths on the time-calibrated phylogeny obtained from Rabosky et al. (2018) using a time-reversible model (REV). Conserved noncoding regions were inferred by comparing, for each genomic region, the neutral model to a conserved model defined by scaling the neutral model by a scaling factor (“rho”) using PhastCons with the following commands: --target-coverage 0.25 --expected-length 12 --rho 0.4 (Siepel et al. 2005). Finally, we used phyloP to identify conserved genomic regions that may have experienced acceleration in sequence divergence in branches where transitions to viviparity are inferred to have occurred (Siepel et al. 2005; Pollard et al. 2010; Hubisz et al. 2011) (fig. 1). To do this, for every predicted conserved noncoding region, we compared the neutral model estimated from 4-fold degenerate sites to a model where we specified acceleration at two foreground branches (fig. 1) using a likelihood ratio test and the following parameters: --msa-format MAF --method LRT --mode ACC. All *P* values were corrected for multiple testing using the Bonferroni–Hochberg procedure.

### Enrichment Analysis for Genomic Elements Associated with Viviparity

To determine the putative functions of genes, we used eggNOG-mapper to annotate genes and PANTHER to perform gene ontology for biological processes using human and zebrafish reference databases for protein-coding genes and genes nearby noncoding elements (Huerta-Cepas et al. 2017; Mi et al. 2017). We also performed network topology-based analysis for genes with 1) convergent amino acid changes, 2) gene-wide convergent sequence change, and 3) noncoding sequence showing convergent sequence evolution. We performed network topology analysis using a zebrafish background gene set via WebGestalt (Liao et al. 2019). We also looked for protein-protein interactions using STRING, with zebrafish as a background gene set (Szklarczyk et al. 2015). We discuss both significant (FDR < 0.05) and nonsignificant enrichments in the Results section.

To test whether noncoding regions showing evidence of accelerated evolution in foreground branches also showed evidence of being putatively regulatory elements, we used Analysis of Motif Enrichment (AME) (Bailey et al. 2009; McLeay and Bailey 2010) to determine whether these regions showed significant enrichment of binding motifs. Binding motifs were detected using curated vertebrate transcription factor databases and compared with randomized sequences using Fisher's exact test (McLeay and Bailey 2010). Since in silico approaches cannot readily and reliably detect genes that are regulated by putative regulatory elements, we followed the approach reported in Sackton et al. (2019) and assumed that putative regulatory elements in *cis* may regulate genes closest to them. To determine genes closest to accelerated putative regulatory elements, we used the BEDTools “closest” function and surveyed the number of accelerated putative regulatory elements nearby each gene (Quinlan and Hall 2010). Here, neighboring genes are loosely defined as the closest gene to a nearby element. Since the criteria that conserved, noncoding regions regulate their closest neighboring gene may be too relaxed, we also report results restricting accelerated, noncoding regions to nearby genes within a 5 kb vicinity.

## Results

### Genome Assembly and Annotation

Genomes assembled in this project, including raw reads and annotation, are available from National Center for Biotechnology Information (NCBI) using the accession codes in supplementary table S1, Supplementary Material online. Metrics for de novo genome assemblies and publicly available genomes are summarized in supplementary tables S2 and S3, Supplementary Material online.

### Reconstructing the Molecular Phylogeny of Cyprinodontiformes

The species tree generated (supplementary fig. 1, Supplementary Material online) has an almost identical topology to the pruned tree obtained from Rabosky et al. (2018) (fig. 1) with two exceptions. First, our tree inferred *G. multiradiatus* as being more distantly related to *G. atripinnis* and *X. captivus*, as opposed to *X. resolanae* as seen in Rabosky et al. (2018). Secondly, *Poecilia formosa* and *Poecilia latipinna* form a species pair in the species tree inferred in this study, which is inconsistent with both Rabosky et al. (2018) and Warren et al. (2018). In both cases of phylogenetic disagreement, branches in our species tree show high levels of gene discordance (33.13% and 48.19%, respectively) (supplementary fig. 1, Supplementary Material online). However, these ambiguous branches are both background branches so are unlikely to impact our analyses. We used the pruned tree from Rabosky et al. (2018) for all subsequent analyses (fig. 1).

### No Significant Evidence for Genome-Wide Convergent Evolution at the Amino Acid Level

In total, 17,572 orthologous protein alignments were generated and filtered, all containing data for each of the 21 species. We found that 2,038 convergent amino acid changes occurred in foreground branches where viviparity is inferred to have evolved. However, these are fewer than those expected under empirical null models. In both empirical null models, foreground branches were randomized along the tree, so that these represented branches that were phenotypically and phylogenetically distinct. The number of convergent amino acid changes observed was 9,018 and 13,937 in the first and in the second empirical null models, respectively. Hence, our data show no evidence for an excess in genome-wide convergent evolution on foreground branches associated with shifts to viviparity. This relates exclusively to the quantity, not to the “quality” of the changes, and although we found that signals of apparent convergence at single amino acid sites are phylogenetically widespread and not restricted to foreground branches, a subset of these genes may nevertheless be consistently linked to the emergence of viviparity but not numerous enough to give a signal in such tests. To explore this, we examined the functions of genes showing evidence of convergent amino acid changes in foreground branches, by performing gene ontology overrepresentation analysis for biological processes against human and zebrafish backgrounds. Using the human background data set, we found an enrichment of genes in important signaling pathways known to be involved in embryogenesis, namely Wnt signaling (fold enrichment = 3.68; FDR = 3.25e^−02^) and Hippo signaling (fold enrichment = 12.49; FDR = 3.79e^−02^) (see reviews; Steinhart and Angers 2018; Davis and Tapon 2019) (supplementary fig. 2*a* and table S4, Supplementary Material online). Using the zebrafish background, we found an enrichment of terms involved in embryogenesis, for example, embryonic viscerocranium morphogenesis (fold enrichment = 4.03; FDR = 4.97e^−02^) and embryonic organ morphogenesis (fold enrichment = 2.34; FDR = 4.56e^−02^) (supplementary fig. 2*b* and table S5, Supplementary Material online). Additionally, we found no significant protein–protein network annotations after correcting for multiple testing, but note relevant annotations include embryonic pattern specification (unadjusted *P* = 0.0167), hematopoietic stem (unadjusted *P* = 0.0086), and progenitor (unadjusted *P* = 0.0167) cell differentiation. We also found evidence of convergent amino acid evolution (FDR < 0.05) in estrogen receptor 1 (GPER1), prolactin (PRL), and luteinizing hormone/choriogonadotropin receptor (LHCGR), suggesting rewiring of hormonal regulation associated with the evolution of viviparity. Additionally, we found evidence of a convergent amino acid change in CD74, a gene critically important in the MHC II pathway and for the recognition of non–self-peptides. In both pipefish and seahorses (Roth et al. 2020), convergent exon loss and divergence of exons in CD74 were associated with the evolution of “male pregnancy.” Finally, we tested the overlap between 115 core placental genes identified across mammals from Armstrong et al. (2017) and genes showing convergently evolving amino acids in our analyses. Only two genes, XBP1 and P4HA1, were found to overlap in both data sets. Further functional studies are required to fully characterize the role of convergent evolution in the structure and function of adaptive immune responses in viviparous Cyprinodontiformes.

As well as searching for convergent amino acid changes, we also assessed relative and correlated rates of protein evolution with phylogenetic switches to viviparity, that is, proteins that changed in evolutionary rate alongside viviparity. We found that 532 genes showed a correlation (*P* < 0.05) between relative rate of protein evolution and the evolution of viviparity, though none were significant after correcting for multiple testing (supplementary table S6, Supplementary Material online). Of these 532 genes, 310 showed slower evolutionary rates at foreground compared with background branches and 222 faster evolutionary rates. However, when compared with empirical null distributions, we again failed to find a significant excess of genes with correlated sequence divergence in our experimental group compared with the controls (ANOVA: df = 3, *F* = 1.8027, *P* = 0.2862).

**Fig. 2.**
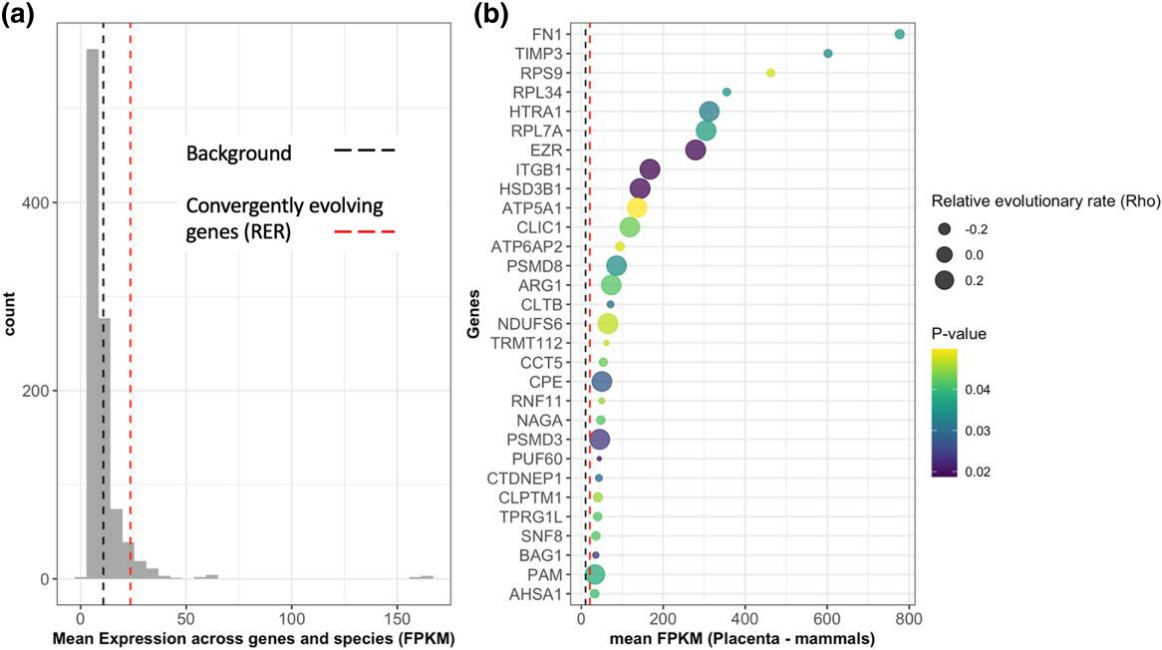
Genes with correlated sequence divergence in branches where transition to viviparity or internal gestation occurred. (*a*) Empirical null distribution of control gene set, with black dashed line showing mean mammalian placental expression across all genes and red dashed line showing mean expression for convergently evolving genes. (*b*) The top 30 genes with correlated sequence divergence ordered by mean mammalian placental expression (FPKM). Unadjusted *P* value indicates significance of relative evolutionary rate.

In order to examine the functions of these 532 genes further, we surveyed mean expression across the placentae of 14 mammalian species and found that 254 genes (with annotations) showed nonnegligible expression with mean FPKM > 1 (*χ*^2^ = 1.0827, df = 1, *P* value = 0.2981). Additionally, those that show accelerated or conserved relative evolutionary rates in branches where viviparity evolved also show higher than average mammalian placental expression compared with both a control gene set (*W* = 163,083, *P* value = 0.0076) and to the background mammalian placenta expression data (*W* = 3,117,536, *P* value < 2.2e^−16^) (fig. 2*a*). Genes showing appreciable expression in placental mammals and correlated sequence divergence include fibroblast growth factors, tyrosine-protein kinase (JAK1), myogenic factor 6 (MYF6), fibronectin (FN1), integrin beta-1 (ITGB1), insulin receptor substrate 1 (IRS1), and notably, SMAD2, a downstream protein in the transforming growth factor β signaling family (fig. 2*b* and supplementary fig. 2, Supplementary Material online). In mammals, members of transforming growth factor β signaling family have been shown to be involved in ovulation (Lee et al. 2007), decidualization (Lee et al. 2007; Fullerton et al. 2017), implantation (Clementi et al. 2013; Peng, Monsivais, et al. 2015), and placentation (Peng, Fullerton, et al. 2015).

**Fig. 3.**
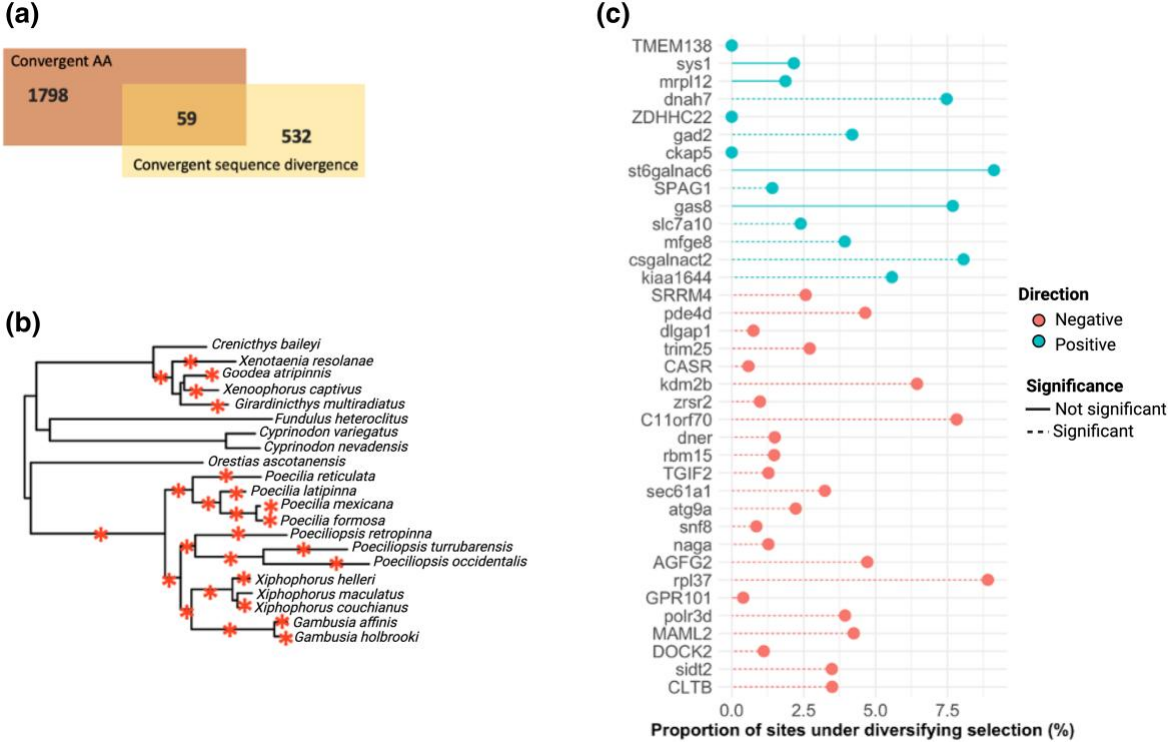
(*a*) Overlap between genes with convergent amino acid substitutions and convergent evolutionary rates. (*b*) The phylogenetic tree of Cyprinodontiformes marked with asterisks denoting branches that were individually tested for positive selection. (*c*) Estimates of proportion of sites under diversifying selection for genes showing both convergent amino acid substitutions and convergent evolutionary rate. Genes with dashed lines are those with evidence of positive selection in at least one branch. Genes are categorized by whether they show constrained or accelerated rates compared with background branches in RER analysis.

By overlapping the results from the 2 previous analyses, we identified 59 genes that showed evidence of both 1) a convergent amino acid change at foreground branches and (2) a significant association between relative evolutionary rate and the trait change to viviparity on foreground branches (fig. 3*a*). To determine whether positive selection has acted on any of these, we conducted gene-wide and branch–site tests using BUSTED and aBSREL (Murrell et al. 2015; Smith et al. 2015). Neither test revealed evidence of positive selection in any of the genes at branches involving switches to viviparity. However, in total, 48 of the 59 genes show evidence of positive selection on at least 1 internal or terminal branch leading to a viviparous lineage and in at least 1 site. To confirm this, aBSREL was used to detect evidence of positive selection on branches leading to viviparous lineages (fig. 3*b*). This identified the same 48 (out of the 59) genes as showing evidence of positive selection (*P* < 0.05). In order to differentiate between genes showing differences in patterns of episodic selection at foreground branches relative to background branches in the phylogeny, we used a modified version of BUSTED to test for 1) episodic selection on foreground branches, 2) background branches, and (c) whether background and foreground branches have shared evolutionary rate distributions. We found, after multiple testing correction, 44 of the 59 genes showed evidence of positive selection on the 2 foreground branches of interest, 54 of the 59 genes also showed evidence of positive selection across background branches (phylogeny-wide positive selection), and only 7 genes showed any evidence of differences in evolutionary rates between foreground and background branches (supplementary table S7, Supplementary Material online). These results indicate most genes showing convincing evidence of convergent evolution also show evidence of phylogeny-wide positive selection, as opposed to positive selection only on foreground branches associated with the evolution of viviparity. Finally, we found no genes that showed evidence of relaxed selection and only two genes—Delta and Notch-like epidermal growth factor-related receptor (DNER) and dedicator of cytokinesis 2 (DOCK2)—showed an intensification of selection in branches where viviparity evolved in Cyprinodontiformes (table 1).

**Table 1.** Nineteen Genes Showing Evidence of Convergent Molecular Evolution, Positive Selection, and Which Are Expressed in Mammalian Placentas and in the Maternal Follicles of Two *Poeciliopsis* Species.

Gene Name	*P* Value (BUSTED)	*P* Value (RELAX)	Found in Mammalian Placentas	Found in Maternal Follicle of *Poeciliopsis* Species	Evidence for Intensification or Relaxation of Selection?
CSGALNACT2	0.0029	0.0672	Yes	Yes	No intensification or relaxation
DOCK2	<0.0001	<0.0001	Yes	Yes	Intensification
ATG9A	<0.0001	0.302	Yes	Yes	No intensification or relaxation
MAML2	<0.0001	0.6401	Yes	Yes	No intensification or relaxation
DNER	0.0001	0.0004	Yes	Yes	Intensification
SIDT2	<0.0001	0.4686	Yes	Yes	No intensification or relaxation
RBM15	<0.0001	0.8877	Yes	Yes	No intensification or relaxation
RPL37	0.0019	0.2578	Yes	Yes	No intensification or relaxation
KDM2B	<0.0001	1	Yes	Yes	No intensification or relaxation
CKAP5	0.0217	0.0323	Yes	Yes	Relaxation
DNAH7	<0.0001	0.281	Yes	Yes	No intensification or relaxation
POLR3D	0.0001	0.019	Yes	Yes	Intensification
PDE4D	<0.0001	0.1176	Yes	Yes	No intensification or relaxation
TRIM25	0.001	0.3251	Yes	Yes	No intensification or relaxation
CLTB	<0.0001	0.0187	Yes	Yes	Intensification
SPAG1	<0.0001	0.6066	Yes	Yes	No intensification or relaxation
SLC7A10	<0.0001	0.6595	Yes	Yes	No intensification or relaxation
SNF8	<0.0001	0.1004	Yes	Yes	No intensification or relaxation
NAGA	0.0027	0.012	Yes	Yes	Relaxation

Despite showing some evidence of both positive selection and convergent molecular evolution, the 59 genes found here may be evolving due to selective pressures for traits that are correlated with the evolution of viviparity (e.g., attributes that, although not responsible for any “viviparity trait,” may nevertheless facilitate the evolution of viviparity). To consider this, we examined whether genes among those 59 were expressed in relevant tissues. Expression data of maternal follicles of 2 species within the *Poeciliopsis* clade (*P. retropinna* and *P. turrubarensis*) are available (Guernsey et al. 2020) and also for placental tissue of 14 mammal species (Armstrong et al. 2017). In mammalian placental tissue, 31 of the 59 genes showed nonnegligible expression (FPKM > 1) (*χ*^2^ = 0.15254, df = 1, *P* value = 0.6961). (supplementary fig. 4, Supplementary Material online). In the *Poeciliopsis*, 21 genes showed nonnegligible expression in either species (*P. retropinna* FPKM > 3.2877, *P. turrubarensis* FPKM > 6.1751, following cutoffs from Guernsey et al. 2020), and these genes also showed concordant nonnegligible expression in mammals.

Altogether, 19 genes (32%) showed 1) a convergent amino acid change, 2) an association between relative evolutionary rate and convergent switches to viviparity, 3) evidence of positive selection on lineages where viviparity has evolved, 4) appreciable expression in either of the maternal follicles of 2 *Poeciliopsis* species, and 5) appreciable expression in the placenta of 14 mammals (table 1). These include DOCK2, which is known to activate RAC1/RAC2 genes required for implantation (Kunisaki et al. 2006; Grewal et al. 2008), and TRIM25, a gene involved in innate immune response against viral infection, mediating estrogen action and whose downregulation during embryogenesis in medaka has been shown to result in apoptosis (Gack et al. 2007; Dong et al. 2012; Zhang et al. 2015).

### Conserved Noncoding Elements Are Convergently Accelerated in Viviparous Lineages

Alongside protein-coding genes, regulatory regions can drive convergent evolution of phenotypes and may be under less functional constraints (Carroll 2008). To identify putative cis-regulatory elements that evolved concordantly in foreground branches, we extracted conserved noncoding regions using a neutral model where substitution rates were estimated from 4-fold degenerate sites and then tested whether any conserved noncoding region showed acceleration of sequence divergence at foreground branches where the transition to viviparity occurred. We found 733,850 conserved noncoding regions evolving significantly slower than 4-fold degenerate sites across the alignment. After correcting for multiple testing, only 245 of these showed significant sequence acceleration at both foreground branches (supplementary table S8, Supplementary Material online). These showed a significant enrichment of transcription factor binding motifs. These transcription factors were enriched in gene ontology terms related to embryonic organ morphogenesis and embryo development ending in birth or egg hatching (supplementary table S9, Supplementary Material online).

**Fig. 4.**
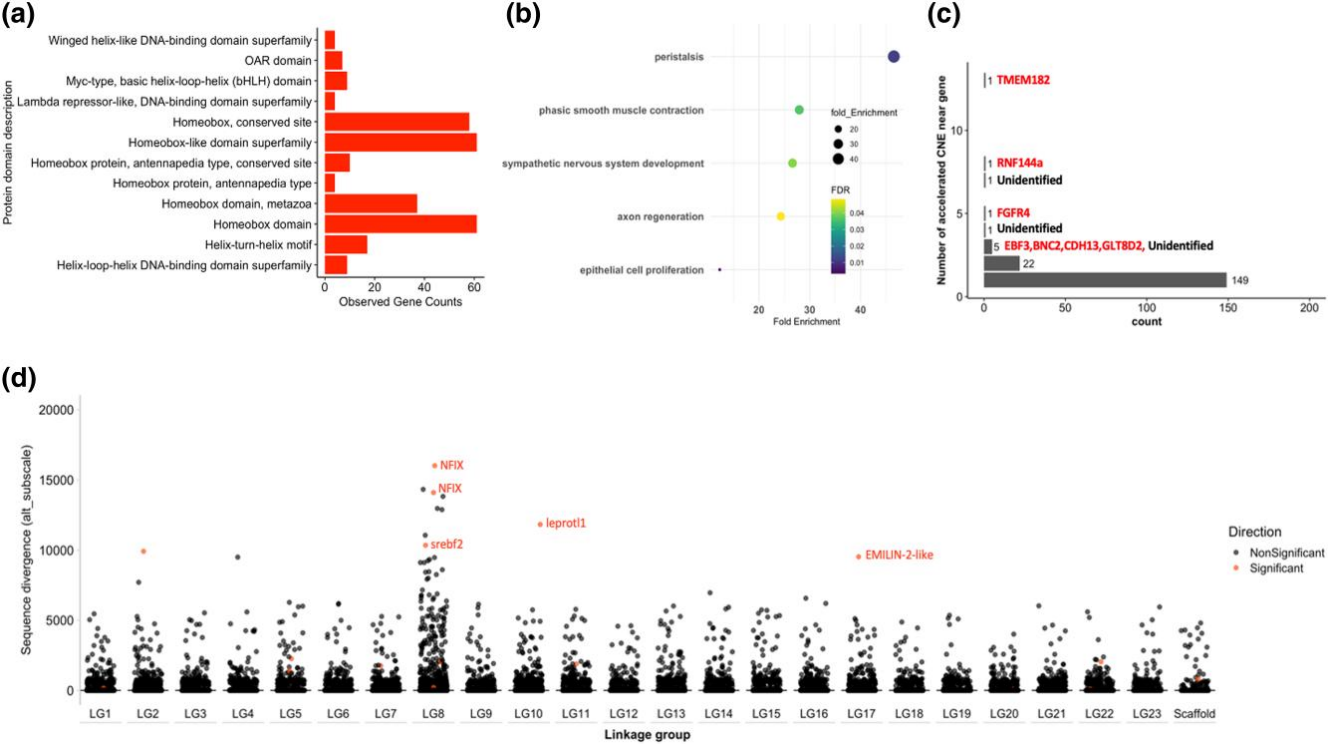
Evidence of convergent evolution in accelerated, conserved, noncoding elements. (*a*) Protein domains of transcription factors associated with putative binding sites in 245 accelerated noncoding elements. (*b*) Gene ontology (biological terms) for genes nearby accelerated noncoding elements. (*c*) Number of accelerated conserved, noncoding elements nearby each gene. Genes with more than five elements nearby are labeled. (*d*) Sequence divergence in foreground branches in conserved, noncoding elements shown across scaffolds arranged by linkage group (based on guppy genome). Noncoding elements in red show significant (FDR < 0.05) acceleration in branches where the transition to viviparity occurred.

To determine what genes may be regulated by the 245 accelerated noncoding elements, we extracted the closest neighboring genes to each accelerated noncoding element. We found 181 genes neighboring at least 1 accelerated noncoding element, with 32 genes showing more than 1 accelerated noncoding element nearby. However, when restricting accelerated noncoding elements to genes within a 5 kb vicinity, we found only 42 genes with at least 1 accelerated noncoding element nearby (supplementary table S10, Supplementary Material online). Genes showing a higher density of accelerated noncoding elements nearby include RNF144a, an E3 ubiquitin ligase, and FGFR4, a fibroblast growth factor receptor. To examine potential functions of all 181 genes, we performed gene ontology analysis of biological and molecular terms and found a significant enrichment of terms related to synapse development, development of the sympathetic nervous system, neuron development, and telencephalon development (fig. 4*b*). Topology-based network analysis of genes nearby accelerated noncoding elements showed significant enrichment of gene expression change (unadjusted *P* = 0.0004) and mesoderm development (unadjusted *P* = 0.0013) annotations, but these enrichment annotations were not significant after multiple testing. Additionally, we found significant enrichments for transcription factors and genes with both E-box (enhancer box) domains and HMG-box domains (fig. 4*a*). These genes include GATA3, SREBF2, NEUROG1, TCF4, POU3F3, and PRXX1. Altogether, motif enrichment and gene ontology analyses suggest transcriptional changes associated with brain development evolved alongside the evolution of viviparity.

### Exploring the Potential Influence of Incomplete Lineage Sorting

To explore the potential impact of ILS in our convergence analysis, we computed gene concordance factors using 17,572 gene trees inferred from orthologous protein alignments and the species tree. We first point out that for our two branches of interest leading to the transition to viviparity, 97% of all gene trees contain the species tree branch leading to viviparous Poecilidae, and 98% of all gene trees contain the species tree branch leading to Goodeinae. These results indicate that relatively few genes considered in our analysis are likely to be discordant in our foreground branches. However, to be conservative, we assess gene concordance in genes with evidence of convergent evolution via 1) convergent amino acid changes and 2) gene-wide convergent evolution. We found no significant difference in gene concordance between convergently evolving genes and all other genes (*Kruskal–Wallis* test: *H* = 52.019, df = 51, *P* = 0.434).

Tests for molecular convergence often rely on a fixed tree topology. We further explored the potential for ILS in our fixed species tree topology to impact the robustness of our results. We used plausible, but underrepresented, alternative topologies that primarily differed in our two foreground branches of interest. Specifically, using these alternative topologies, we reassessed relative protein evolution in our foreground branches, where hypothesized transitions to viviparity occurred, relative to background branches across the phylogeny using RERconverge. Repeating our analyses using 2 alternative topologies, we found 408 (in alternative topology A) genes and 731 genes (in alternative topology B) showed evidence of correlated sequence change with the evolution of viviparity. Our reanalysis also revealed variation in the number of genes with correlated sequence change that were robust to ILS, assessed via the overlap between convergently evolving genes under the fixed species tree topology and alternative topologies. Alternative topology A showed considerable overlap with the fixed species tree topology (246 overlapping genes), whereas the second showed little overlap (66 overlapping genes). Thirty-one genes were found to overlap between all three topologies, suggesting that only a few genes evolving under convergent evolution are robust to ILS. Among these, only seven were found to have reliable annotations and only one gene, *DNER*, was found to also show consistent signals of molecular convergence across all tests.

To understand the potential effect of hemiplasy (due to ILS alone), we estimated the likely number of genetic changes associated with viviparity by simulating 10^8^ loci along the inferred Cyprinodontiformes species tree. Using HeIST, we converted branch lengths (in substitutions per site) to coalescent units using gene concordance estimates, and the regression approach is detailed in Hibbins et al. (2020). We found that for focal cases—where simulated nucleotide character states (denoted either 0/1 for ancestral and derived states, respectively) match character traits in the species tree—a scenario where 1) all viviparous taxa are grouped together in a single monophyletic clade and where 2) a transition to viviparity is explained by a single mutation is more common (41/79 of focal loci) than homoplasy (38/79 of focal loci). These results suggest that under the assumption that transitions to viviparity are underpinned by a simple genomic architecture, there is an almost equal probability that viviparity in Cyprinodontiformes may be explained by a single transition (ancestral polymorphism) rather than two.

## Discussion

Comparative studies of vertebrate viviparity, stimulated by development of phylogenetic comparative methods and growing evidence of molecular convergence in general (Smith et al. 2020), are beginning to ask if there is a common genetic underpinning of viviparity (Lynch et al 2008; Kin et al 2016). Some have shown remarkable convergent evolution of genes and pathways involved in independent acquisitions of internal gestation across both closely and distantly related species (Roth et al. 2020; Recknagel et al. 2021; van Kruistum et al. 2021); however, other studies exploring phenotypic and molecular convergence of viviparity suggest that species can evolve striking parallel evolution by redeployment of different genes (Foster et al. 2022). Here, we have considered the role of molecular convergence in the repeated evolution of viviparity in Cyprinodontiformes. We found no evidence for an excess of genome-wide molecular convergence in amino acid changes or in rates of protein evolution in the convergent evolution of viviparity. However, we identified a small subset of candidate genes that show evidence of convergent evolution and are homologous to genes essential in mammalian viviparity. Our results therefore suggest viviparity could repeatedly involve some key genes but is not strongly associated with a genome-wide signal of convergence in the Cyprinodontiformes and may also incorporate independent genetic changes within this group.

### The Evolution of Viviparity Is Not Associated with a Significant Excess of Genome-Wide Molecular Convergence

An important source of support for convergent evolution in comparative genomic analysis is finding convergent amino acid substitutions or convergent sequence divergence in independent lineages that are greater than expected by chance (Zhang 2003; Storz 2016). However, the detection of genome-wide molecular convergence has proved contentious. For example, comparative surveys identifying convergence in mammalian lineages that independently evolved echolocation were shown to have fewer parallel substitutions than comparisons between echolocating and nonecholocating mammals (Parker et al. 2013; Thomas and Hahn 2015; Zou and Zhang 2015). Similarly, behavioral and morphological phenotypic convergence in anole lizards is not associated with parallel substitutions or rates of amino acid change, suggesting either 1) difficulty in detecting genome-wide convergence or 2) that the same loci are not repeatedly recruited (Corbett-Detig et al. 2020). Similarly, in a comparison of oviparous and viviparous lizards, evidence for convergent amino acid change was minimal, with most consistent differences observed in gene expression (Gao et al. 2019). Independent acquisitions of placentotrophy in Poecilidae showed a preponderance of protein-coding genes undergoing shifts in sequence divergence in placental species compared with nonplacental species (van Kruistum, et al. 2021). In our study, we did not find a higher incidence of convergence at any level examined—in fact, we found a higher incidence of expected molecular convergence in our null models.

However, a small subset of genes in each of our analyses show striking resemblance to genes critically important for mammalian viviparity, and they belong to gene families previously implicated in viviparity-related adaptations. For example, we found evidence of convergent amino acid substitution in PRL. In Eutherian (placental) mammals, PRL is produced throughout pregnancy, stimulates cell proliferation and differentiation, and maintains production of progesterone and relaxin (Soares et al. 2007). Additionally, PRL expression was previously observed in the maternal follicle of matrotrophic species of *Poeciliopsis*, but not in lecithotrophic species of *Poeciliopsis*, suggesting a potential role for PRL in the evolution of matrotrophy in Poecilidae and Goodeinae (Guernsey et al. 2020). We also found evidence of convergent evolution in genes critical to adaptive immunity that are also associated with the evolution of viviparity. Changes to adaptive immunity in viviparous lineages may be responsible for maternal immunotolerance toward developing embryos. In gestating seahorses and pipefish, there is similar loss of genes related to the MHC II pathway and rapid sequence evolution of CD74, an invariant chain of MHC II involved in preventing premature binding (Roth et al. 2020). Here, we found evidence of convergent substitution in a gene involved in the MHC II pathway in viviparous lineages, though it is unknown whether this substitution is neutral or under selection.

### No Evidence of Positive Selection on Branches Associated with the Transition to Viviparity

We assessed sequence divergence associated with viviparity and compared signatures of convergence with placental expression data across mammals. We found that a number of genes showing concordant sequence divergence show elevated expression in all mammalian placentas, suggesting that genes implicated in mammalian pregnancy may be repeatedly recruited in nonmammalian viviparity. These include HTRA1, EZR, ITGB1, and HSD3B1 which have roles in fetal adhesion, fetal growth, invasion of endometrial stromal cells, and modulation of progesterone (Ornek et al. 2008; Shimodaira et al. 2012; Burkin et al. 2013; Nishimura et al. 2014). Some genes may be housekeeping ones with ubiquitous expression that are involved in general cellular maintenance and metabolism. For example, we identify a number of ribosomal proteins (RPS9, RPL34, and RPL7A) that are highly expressed across mammalian placentas and show signals of concordant sequence divergence.

We found 59 genes with evidence of both a convergent amino acid change and correlated sequence divergence. Although some of these show evidence of positive selection in at least one branch in the phylogeny, none showed evidence of positive selection in both branches associated with the transition to viviparity. These results mirror patterns of convergent evolution of placentae in Poecilidae (van Kruistum, et al. 2021). Similarly, an analysis of marine mammals found that very few genes showing concordant sequence divergence also showed evidence of positive selection (Chikina, Robinson and Clark 2016). Instead, genes showing accelerated sequence divergence in marine mammals were probably subject to relaxed constraints (Chikina, Robinson and Clark 2016). Here, we found no evidence of relaxed selection in the 59 genes on branches associated with the transition to viviparity. Since all 59 genes were tested for positive selection in branches associated with the transition to viviparity, as well as all internal and terminal branches leading to viviparous species, correcting for multiple testing may have raised the bar to detect potential signals of positive selection only on branches associated with the transition to viviparity.

### Can Biological Confounds Explain Independent Transitions to Viviparity in Cyprinodontiformes?

Our analyses are necessarily constrained in power by only including two independent transitions to viviparity in our samples. As well as statistical power, another potential confound in detecting parallel evolution arises due to collateral evolution. Collateral evolution describes the evolution of phenotypic convergence via genetic variation that was either 1) the result of some introgression event or 2) via the involvement of an ancestral polymorphism and ILS (Stern 2013). Here, we addressed the potential for collateral evolution to explain patterns of molecular convergence. To ask whether ILS might impact our results, we conducted gene concordance factors, asking how well each branch in the species tree is represented across gene trees inferred from all protein-coding genes in our data set. In our two foreground branches, we observed high levels of gene concordance indicating most protein-coding genes in our data set show little conflict at branches we repeatedly test for associations with viviparity. Similarly, when considering how hypothetical discordance at these two foreground branches of interest may have affected our analyses by reanalyzing gene-wide convergent evolution under alternative topologies, we still recovered a substantial proportion of genes showing evidence of gene-wide convergent evolution under the species tree topology. These reanalyses suggest our key findings are not likely to be significantly impacted by potential ILS.

Additionally, when simulating hemiplasy under ILS alone, we found an almost equal probability of viviparity in Goodeinae and Poecilidae having a single genetic basis. However, there are a number of important caveats. First, we do not include all independent transitions to viviparity; in particular, we do not include the evolution of viviparity in Anablepidae (Helmstetter et al. 2016), which likely results in underestimating the probability of a homoplastic origin in our analysis. Secondly, the approach used assumes the genetic architecture of simulated traits is monogenic, but a simple genetic architecture is unlikely given the necessary and complex adaptations required for viviparity to evolve. Thirdly, although we simulated a large number of loci, only 79 focal loci matched the species tree and were considered as either hemiplastic or homoplastic. As a result, whether hemiplasy is likely to play a major role in our ability to detect convergent evolution probably remains an open question.

### Accelerated Noncoding Regions Associated with the Evolution of Viviparity Show Evidence of Functionality

Beyond assessing molecular convergence in coding regions, we also sought to identify noncoding regions undergoing accelerated sequence divergence in branches associated with the transition to viviparity. We identified a small number of conserved noncoding regions showing accelerated sequence divergence and nearby genes that may be associated with these regions. These showed an enrichment of transcription factor binding motifs, suggesting functionality of identified accelerated noncoding loci and potential rewiring of gene expression associated with the evolution of viviparity. In particular, some of the genes identified were located nearby multiple accelerated noncoding loci, for example, FGFR4, a fibroblast growth factor receptor with evidence of expression in the human placenta (Anteby et al. 2005). We also detected 149 genes with only a single accelerated noncoding loci nearby with known roles in mammalian viviparity (supplementary table S8, Supplementary Material online). For example, GATA3 has previously been identified as a critical and conserved component of placental development in mammals (Home et al. 2017; Gerri et al. 2020). However, when genes were considered together via gene ontology analysis, we found an enrichment for genes with a potential role in mediating the development of the central nervous system. Although these analyses do not clearly implicate particular pathways, they suggest a cohort of genes with potentially broad functions which may have undergone changes in gene expression during the evolution of viviparity in goodeids and poecilids.

## Conclusions

The study of convergent evolution of genes underlying traits such as complex morphologies often highlights contrasts between consistent changes in cohorts of consistent genes versus redeployment of independent gene networks. Our analyses indicate that this might be a somewhat artificial distinction. We find both a lack of a strong quantitative signal of concerted parallel changes in the evolution of viviparity in the Cyprinodontiformes but convincing evidence of the consistent involvement of a few genes important to the evolution of viviparity and internal gestation across these and additional vertebrates. More independent transitions to viviparity will help resolve statistical issues in detecting consistent gene changes, but our study suggests more attention to potential biological confounds such as hemiplasy and introgression is also needed.

## Supplementary Material

Supplementary data are available at *Molecular Biology and Evolution* online.

## Supplementary Material

msad208_Supplementary_Data

## Data Availability

Genomes assembled in this project, including raw reads and annotation, are available from NCBI using the accession codes in supplementary table S1, Supplementary Material online. Scripts used to generate the assemblies and annotation can be found here: https://github.com/peterthorpe5/fish_genome_assembly. Scripts used in the comparative analysis of convergent evolution can be found here: https://github.com/LeebanY/Convergent-evolution-of-viviparity-in-Cyprinodontiformes.
