# Supplementary material for: Genomic Signatures Associated with Transitions to Viviparity in Cyprinodontiformes
Source: Mol Biol Evol. 2023 Oct 4;40(10):msad208. doi: 10.1093/molbev/msad208 (PMC10568250; doi:10.1093/molbev/msad208)
Supplement: msad208_Supplementary_Data [file msad208_supplementary_data.zip › SupplementaryFigures.pdf]

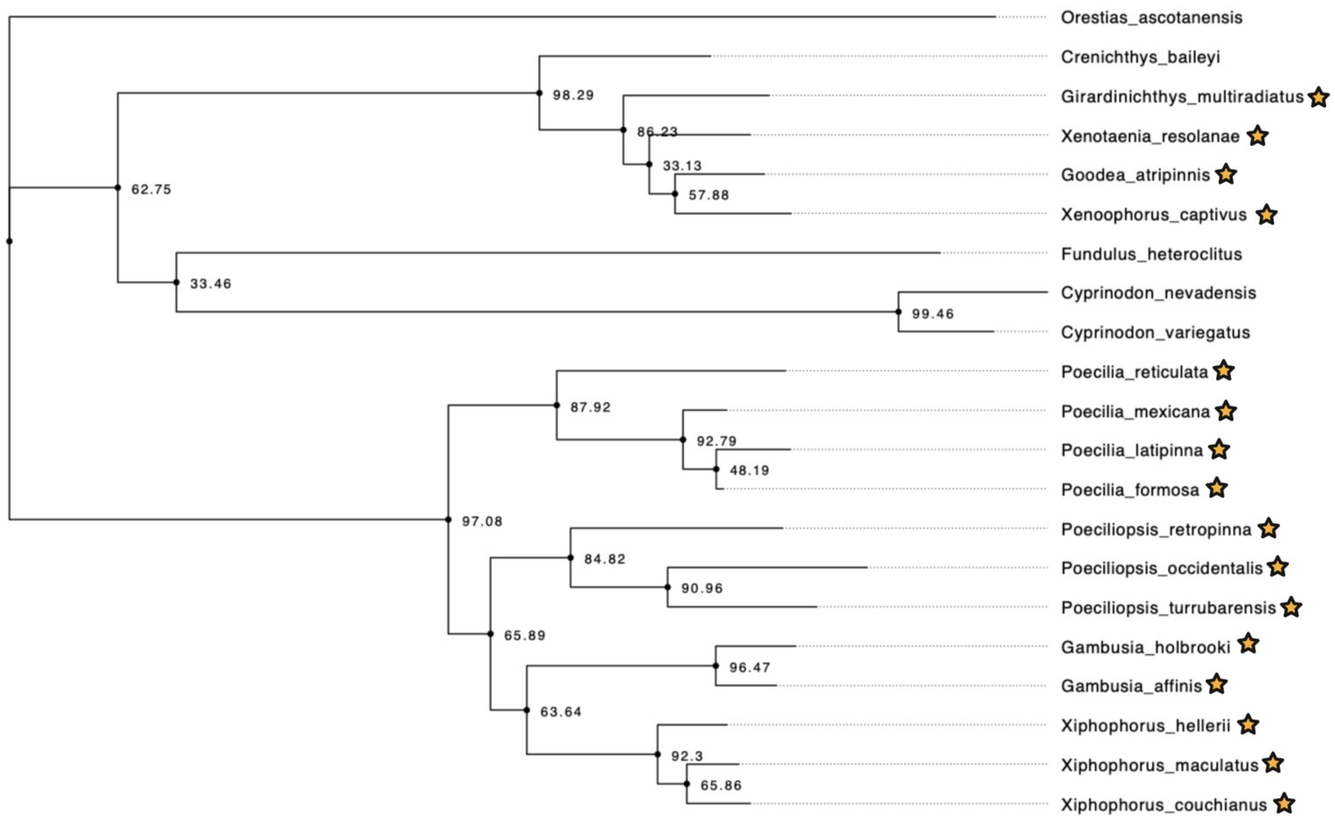

Supplementary Figure 1: Species tree inferred using 1,044 single-copy orthologs. Gene concordance factors (shown for each node) estimated using 16,941 gene trees. Branch length represented by scale bar. Stars represent species that are viviparous, all other species in the tree are oviparous.

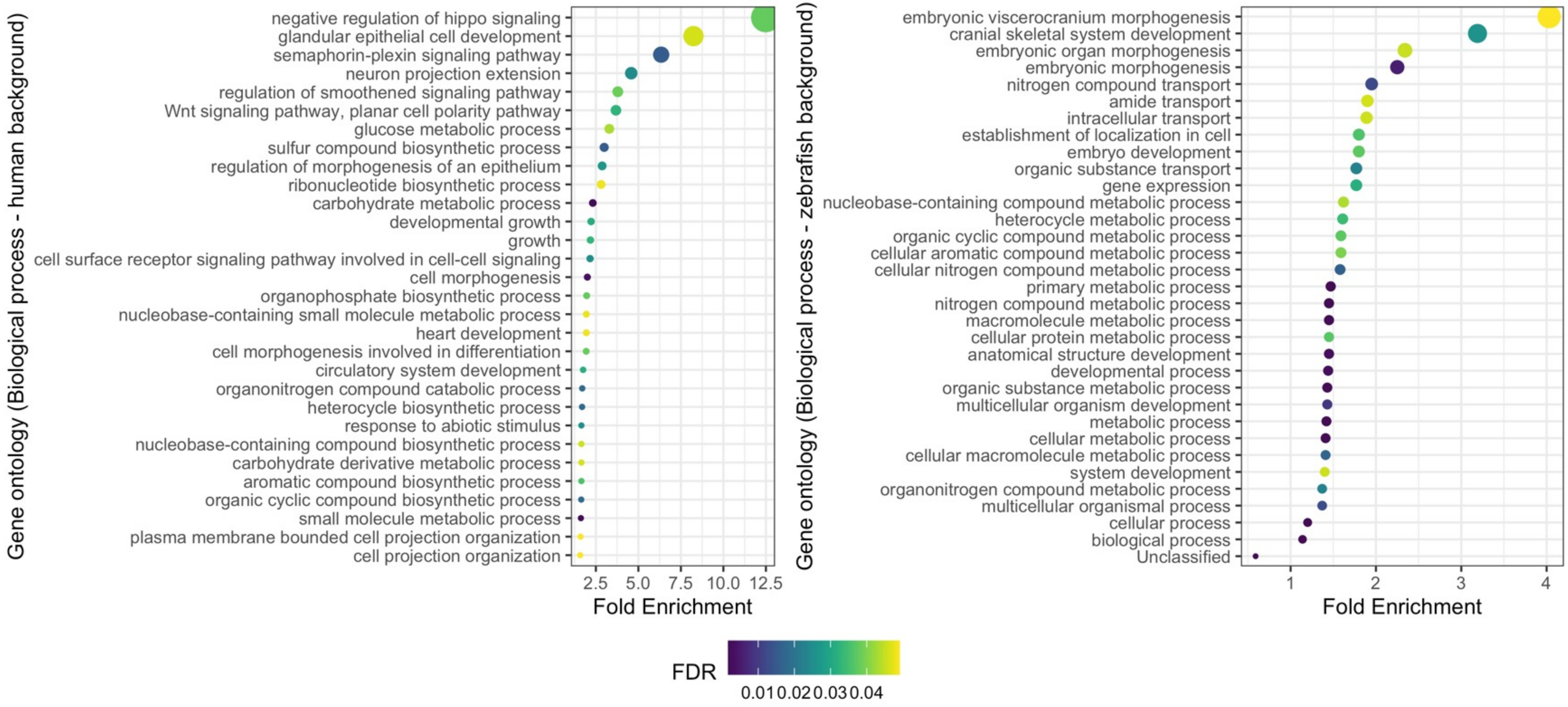

Supplementary Figure 2: Gene ontology for biological processes of genes with a convergent amino acid change in viviparous foreground branches. Enrichment of biological processes against a human (left) and a zebrafish background/database (right) are plotted. Colours in both plots represent false discovery rates and size of points also indicate fold enrichment.

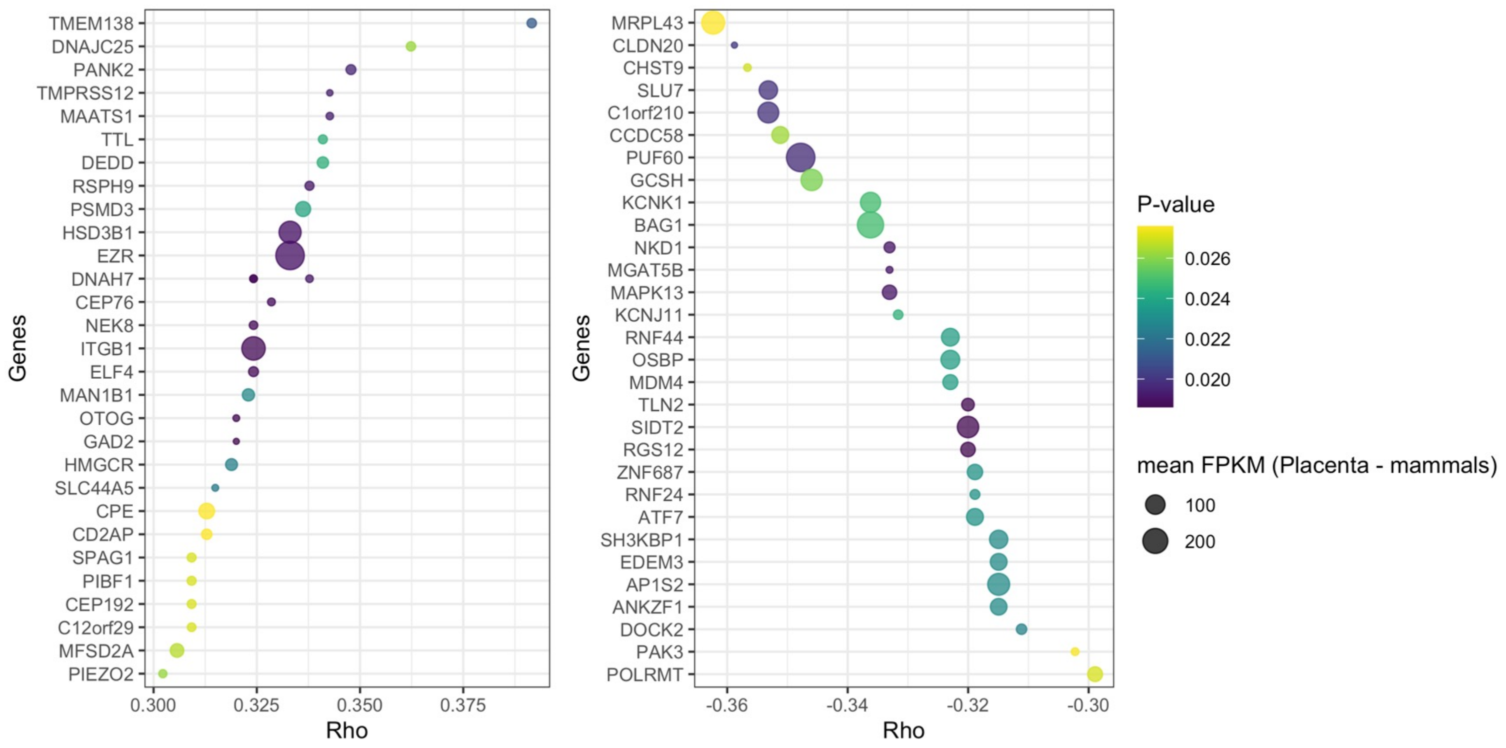

Supplementary Figure 3: Genes undergoing convergent shifts in evolutionary rate in viviparous foreground branches. The top 30 genes with fastest rates of protein evolution (left), and the top 30 genes with slowest rates of protein evolution (right) are shown. Rho indicates the correlation between change in trait (reproductive mode) and relative rate of protein evolution. Here, points are coloured by p-value from the correlation analysis and the size of each point is indicative of mean FPKM (fragments per kilobase per million reads) in placental tissue across 14 mammal species.

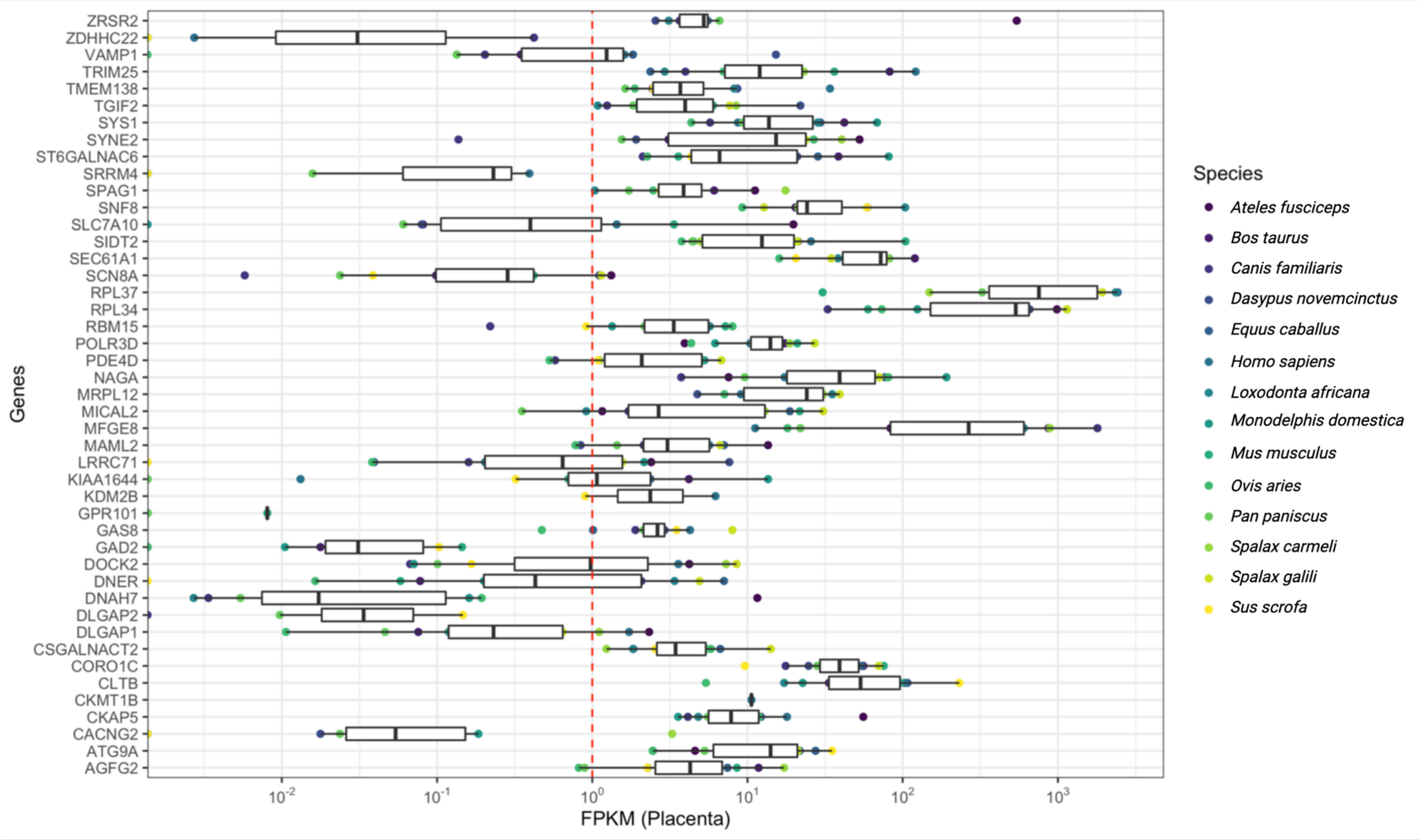

Supplementary Figure 4: Mean placental expression in 14 mammals of genes showing signals of convergent evolution in foreground branches. Expression is represented as mean fragments per kilobase of transcript per million mapped reads (FPKM) for each gene. Coloured points represent mean FPKM for each respective gene and for a given species. Red dotted line indicates FPKM>1 cut-off. X axis is log[10] scaled.
