# Supplementary figures and images for: Genomic Signatures Associated with Transitions to Viviparity in Cyprinodontiformes
Source: Mol Biol Evol. 2023 Oct 4;40(10):msad208. doi: 10.1093/molbev/msad208 (PMC10568250; doi:10.1093/molbev/msad208)

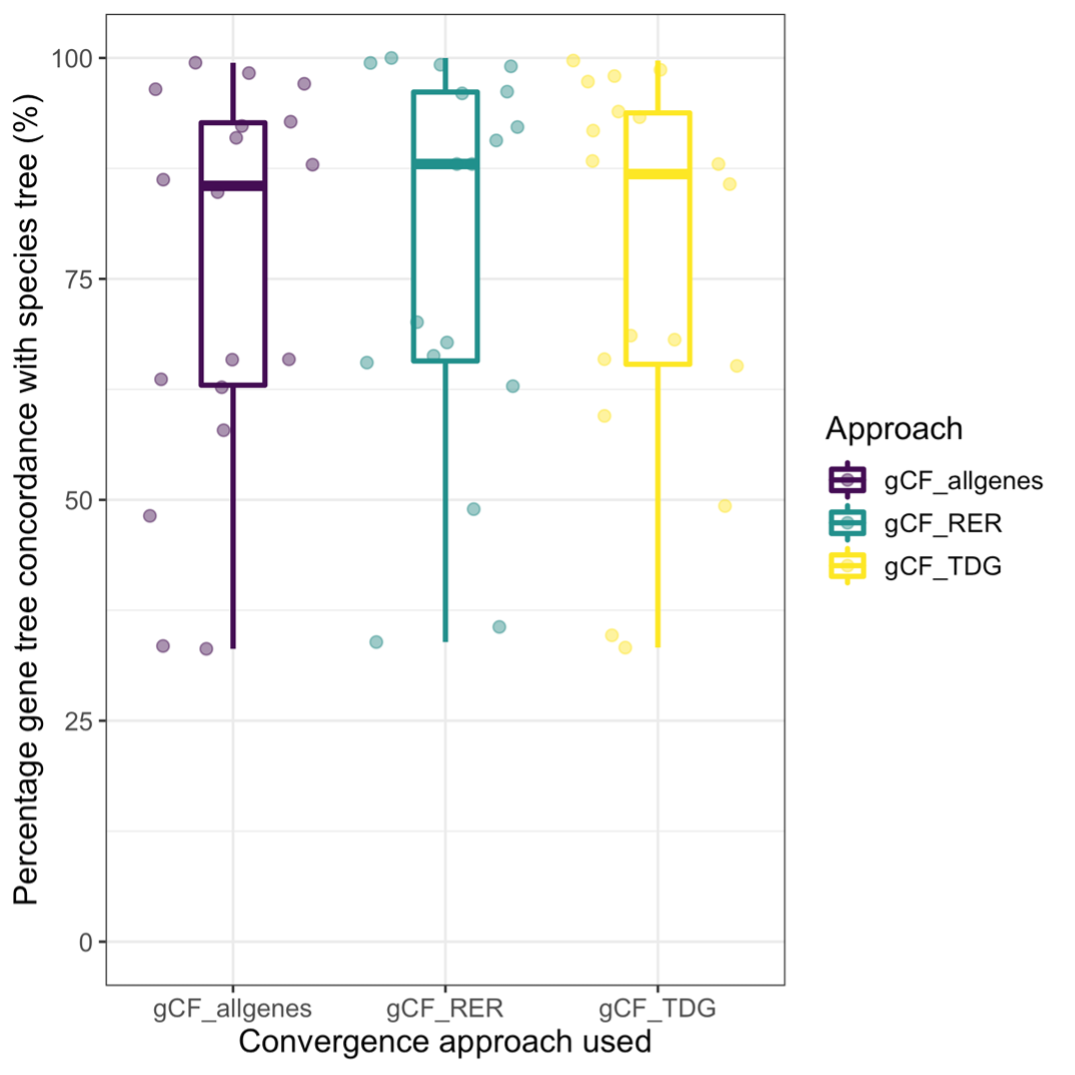

Supplement: msad208_Supplementary_Data [file msad208_supplementary_data.zip › SupplementaryFig5.png]
